# Early Gene Expression in Wounded Human Keratinocytes Revealed by DNA Microarray Analysis

**DOI:** 10.1002/cfg.239

**Published:** 2003-02

**Authors:** Manal A. Dayem, Chimène Moreilhon, Laurent Turchi, Virginie Magnone, Richard Christen, Gilles Ponzio, Pascal Barbry

**Affiliations:** 1 Laboratoire de Physiologie Génomique des Eucaryotes CNRS/UNSA UMR 6097 IPMC F-06560 Sophia Antipolis France; 2 Laboratoire de Biologie et Physiologie de la Peau INSERM U385 Faculté de Médecine Nice F-06107 France; 3 Laboratoire de Physiologie des Membranes Cellulaires CNRS UMR6078 Bat. J. Maetz Villefranche-sur-Mer France; 4 Laboratoire de Physiologie Génomique des Eucaryotes IPMC, 660 Route des Lucioles Sophia Antipolis Valbonne 06560 France

## Abstract

Wound healing involves several steps: spreading of the cells, migration and proliferation. We have profiled gene expression during the early events of wound healing in normal human keratinocytes with a home-made DNA microarray containing about 1000 relevant human probes. An original wounding machine was used, that allows the wounding of up to 40% of the surface of a confluent monolayer of cultured cells grown on a Petri dish (compared with 5% with a classical ‘scratch’ method). The two aims of the present study were: (a) to validate a limited number of genes by comparing the expression levels obtained with this technique with those found in the literature; (b) to combine the use of the wounding machine with DNA microarray analysis for large-scale detection of the molecular events triggered during the early stages of the wound-healing process. The time-courses of RNA expression observed at 0.5, 1.5, 3, 6 and 15 h after wounding for genes such as *c-Fos, c-Jun, Egr1*, the plasminogen activator PLAU (uPA) and the signal transducer and transcription activator STAT3, were consistent with previously published data. This suggests that our methodologies are able to perform quantitative measurement of gene expression. Transcripts encoding two zinc finger proteins, ZFP36 and ZNF161, and the tumour necrosis factor α-induced protein TNFAIP3, were also overexpressed after wounding. The role of the p38 mitogen-activated protein kinase (p38MAPK) in wound healing was shown after the inhibition of p38 by SB203580, but our results also suggest the existence of surrogate activating pathways.


**Comparative and Functional Genomics**
*Comp Funct Genom* 2003; **4**: 47–55.
Published online in Wiley InterScience (www.interscience.wiley.com). **DOI:** 10.1002/cfg.239

**Conference Paper**

### Early gene expression in wounded human keratinocytes revealed by DNA microarray analysis

Manal A. Dayem[1], Chimène Moreilhon[1], Laurent Turchi[2], Virginie Magnone[1], Richard Christen[3], Gilles Ponzio[2] and Pascal Barbry[1]*

[1] *Laboratoire de Physiologie Génomique des Eucaryotes, CNRS/UNSA UMR 6097, IPMC, F-06560 Sophia Antipolis, France*
[2] *Laboratoire de Biologie et Physiologie de la Peau, INSERM U385, Faculté de Médecine, F-06107 Nice, France*
[3] *Laboratoire de Physiologie des Membranes Cellulaires, CNRS UMR6078 Bat. J. Maetz, Villefranche-sur-Mer, France*

**Correspondence to:*
*Pascal Barbry, Laboratoire de Physiologie Génomique des Eucaryotes, IPMC, 660, Route des Lucioles, Sophia Antipolis, 06560 Valbonne, France.*
*E-mail: barbry@ipmc.cnrs.fr*

Received: 13 September 2002
Accepted: 21 November 2002

### Abstract

**Wound healing involves several steps: spreading of the cells, migration and proliferation. We have profiled gene expression during the early events of wound healing in normal human keratinocytes with a home-made DNA microarray containing about 1000 relevant human probes. An original wounding machine was used, that allows the wounding of up to 40% of the surface of a confluent monolayer of cultured cells grown on a Petri dish (compared with 5% with a classical 'scratch' method). The two aims of the present study were: (a) to validate a limited number of genes by comparing the expression levels obtained with this technique with those found in the literature; (b) to combine the use of the wounding machine with DNA microarray analysis for large-scale detection of the molecular events triggered during the early stages of the wound-healing process. The time-courses of RNA expression observed at 0.5, 1.5, 3, 6 and 15 h after wounding for genes such as *c-Fos, c-Jun, Egr1*, the plasminogen activator PLAU (uPA) and the signal transducer and transcription activator STAT3, were consistent with previously published data. This suggests that our methodologies are able to perform quantitative measurement of gene expression. Transcripts encoding two zinc finger proteins, ZFP36 and ZNF161, and the tumour necrosis factor $\alpha$-induced protein TNFAIP3, were also overexpressed after wounding. The role of the p38 mitogen-activated protein kinase (p38MAPK) in wound healing was shown after the inhibition of p38 by SB203580, but our results also suggest the existence of surrogate activating pathways.** **Copyright © 2003 John Wiley & Sons, Ltd.**

**Keywords:** **wound healing; p38 cascade; ERK cascade; microarrays; Egr1; zinc proteins; STAT proteins; SB203580**

### Introduction

After an epithelial injury, wound healing results from a combination of cell growth, proliferation, migration and differentiation, all of these processes being associated with altered expression of specific genes. Although some of these genes are already known, the specific pathways involved in the transduction of the healing signals remain largely to be defined. In mammals, three distinct mitogen-activated protein kinase (MAPK) cascades have been identified: (a) the c-Jun-N-terminal protein kinase (JNK) cascade; (b) the p38 MAP kinase (p38MAPK) cascade; and (c) the extracellular signal-regulated kinase (ERK) cascade. They are known to be involved in the transduction of stress- or injury-induced signals (Jaakkola *et al.*, 1998; Turchi *et al.*, 2002). The activation of MAP kinases has been observed after wounding in cultured cells (Dieckgraefe *et al.*, 1997; Goke *et al.*,

Copyright © 2003 John Wiley & Sons, Ltd.

1998) and entire tissues (Seo *et al.*, 1995; Aronson *et al.*, 1998). The ERK cascade also plays an important role in mediating cell proliferation in response to growth factors. ERK displays a biphasic activation with two peaks, at 10 min and after 2 h (Meloche *et al.*, 1992). The first peak coincides with the monophasic p38 activation (Lee *et al.*, 2000). C-*fos*, the early growth response factor *Egr1* (or *Krox-24*), and other immediate early genes, are activated after a wound (Grembowicz *et al.*, 1999; Martin and Nobes, 1992; Grose *et al.*, 2002; Verrier *et al.*, 1986). Because many of these genes encode transcription factors, they are able to transactivate multiple selected secondary targets.

In order to identify new immediate early genes, we have used a spiral scarificator, that develops a large wound over the surface of a confluent monolayer of cultured cells (Turchi *et al.*, 2002). The two aims of the present study were: (a) to validate a limited number of genes (about 1000 probes) by comparing the expression levels obtained with this technique with those found in the literature; (b) to combine the use of the wounding machine with DNA microarray analysis, in order to detect on a large scale the molecular events triggered during the early stages of the wound-healing process under controlled conditions.

### Materials and methods

### Probe design and microarray printing

The cDNA probes were produced by PCR amplification. The probes were selected from 13440 RefSeq sequences (i.e. confirmed human transcripts), using an automated BLAST parsing program. All probes (i) had a normalized length of 250 bp $\pm$ 19 bp; (ii) had a normalized GC content of 52% $\pm$ 8%; and (iii) were specific for a unique human gene. Probes were selected among ion or solute transport proteins, cytokines and other inflammatory molecules, growth factors, metalloproteases, transcription factors, protein kinases and signal transduction molecules. The final list of the probes spotted on the microarray is available on **http://medlab.ipmc.cnrs.fr/Gene_List.html**. We purified our PCR products using 96-well multiscreen filter plates (MAFNOB 50, Millipore). The arrays were printed on aldehyde-coated glass microscope slides using a robot (SDDC-2, Virtek arrayer). Microarrays were prepared by printing in duplicate PCR amplicons resuspended in 3×SSC, with an average spotting concentration of 200 ng/μl.

### Target preparation and hybridization

#### Keratinocyte primary cultures and wound healing

Human keratinocytes were isolated from healthy neonatal foreskin and grown as confluent monolayers in 100 mm culture dishes as described by Rheinwald and Green (1975).

The wounding was performed using a spiral scarificator, previously described by Turchi *et al.* The cultures were stopped by washing with phosphate buffer saline, followed by a quick freezing to −80 °C, at different times after wounding (30 min, 1 h 30 s, 3 h, 6 h and 15 h). For each time of the kinetic series, non-wounded cells were used as controls. In experiments performed with the p38MAPK inhibitor, 30 μM SB203580 were preincubated with the cells 2 h before wounding.

#### RNA extraction

Cells (5–10 x $\times$ $10^6$) were quickly homogenized in 10 ml 4 M guanidium thiocyanate solution, 25 mM sodium citrate, pH 7.0, 100 mM $\beta$-mercaptoethanol, 0.5% $N$-laurylsarcosine, followed by the addition of 1 ml 2 M sodium acetate, pH 4.0, 8 ml freshly water-equilibrated phenol, and 2 ml chloroform. After 15 min on ice, the samples were centrifuged for 20 min at 7500 $\times$ $g$. The RNA of the upper aqueous phase was precipitated with one volume of isopropanol. The sample was incubated for 1 h on ice, after which the RNA was pelleted by centrifugation for 20 min at 7500 $\times$ $g$. The pellet was washed twice with 70% ethanol, then resuspended in RNase-free water.

#### Cy3- and Cy5-labelled cDNA

The CyDye-labelled first-strand cDNAs were generated with the CyScribe First-Strand cDNA Labelling Kit (RPN 6200, Amersham Pharmacia Biotech). We used Cy3-dCTP and Cy5-dCTP (Amersham Pharmacia Biotech) for a direct labelling. Total RNA (5–10 μg) was used as template. Unincorporated CyDye-nucleotides were removed using the Nucleotide Removal Kit (Qiagen).

Copyright © 2003 John Wiley & Sons, Ltd. 

#### Post-processing

All washes were performed at room temperature, except when noted, with vigorous agitation. Printed arrays were post-processed by washing twice in 0.2% SDS for 2 min, then twice for 2 min in distilled water, followed by 2 min in 95–100 °C distilled water. Arrays were then treated for 5 min in a $NaBH_4$ solution (0.5 g $NaBH_4$ in 150 ml PBS and 45 ml ethanol). The arrays were rinsed three times in 0.2% SDS for 2 min, followed by two washes in distilled water for 1 min. The arrays were then immersed for 30 min in a blocking solution (0.5 g casein, 250 ml PBS, 200 µl Tween 20; I-block, Tropix, Bedford, MA) and finally washed for 2 min in 0.2% SDS, three times for 1 min in distilled water, spun dry, then stored under vacuum.

#### Hybridization

The microarray was covered with 20 µl hybridization buffer (DigEasy Roche) containing the Cy3/5-labelled samples, and was overlain with a glass cover slip (Erie Scientific, Portsmouth, USA). The hybridization was performed overnight at 48 °C. Arrays were washed for 5 min in 1×SSC–0.03% SDS, 5 min in 0.2×SSC, 5 min in 0.05×SSC, 1 min in $H_2O$, 1 min in isopropanol, then spun dry.

### Data collection and analysis

#### Data collection

Arrays were scanned using a confocal gas-laser scanner (ScanArray 5000, GSI Lumonics), and the ScanArray program (LEAD Technologies Imaging Products). The two red and green lasers operated at 633 nm and 543 nm to excite Cy5 and Cy3, respectively. The intensity was measured at 670 nm for Cy5 and 570 nm for Cy3. The two 16-bit Tiff images containing the data from each fluorescence channel were analysed by the QuantArray (2.0.0.0a) program.

#### Centralization

Cy3 and Cy5 dyes differ in their respective incorporations into cDNA (related to their relative affinities for the reverse transcriptase) and their quantum yields. This leads to significant differences in emissions. In order to eliminate dye-dependent differences, a 'dye-swap' protocol was used: in a first microarray, cDNAs derived from wounded and control cells were labelled with Cy3 and Cy5, respectively; in a second 'swap' microarray, cDNAs derived from wounded and control cells were labelled with Cy5 and Cy3, respectively (Kerr and Churchill, 2001). If $Cy3(gene_i)[wounded]$ represents the fluorescence intensity for $gene_i$ in the first slide, and $Cy5(gene_i)[wounded]_{swap}$ represents the fluorescence intensity for the same $gene_i$ in the swapped slide, the specific signal associated with $gene_i$ in RNA derived from wounded cells (called $wounded[gene_i]$) is defined as the geometrical average of the specific intensities in Cy3 and Cy5:

$$\text{wounded}[\text{gene}_i] = \sqrt{\text{Cy3}(\text{gene}_i)[\text{wounded}] \times} \sqrt{\text{Cy5}(\text{gene}_i)[\text{wounded}]_{swap}}$$

This value is independent of cyanine incorporation (not shown).

#### Experimental design

Each time point was independently analysed three times. The keratinocytes used for these three independent time-courses were derived from the same primary culture. Taken together with the two arrays (one array and its swap) hybridized for each experimental condition, data for each time point in the present study were derived from a total of six arrays.

In order to ascertain the quality of hybridization, each array contained 192 internal control spots corresponding to 'spike' control probes. These control DNA probes were spotted at concentrations ranging from 0 to 200 ng/µl and correspond to invertebrate sequences that do not cross-react with any known mammalian genes (corresponding to GenBank Accession Nos X92113, Y16225, and Y16240). Corresponding RNAs were produced by *in vitro* transcription, and the experimental RNAs were reverse-transcribed in the presence of different amounts of the three different 'spike' RNAs. These different amounts of spike RNA resulted in ratios of 1 : 1, 1 : 0 and 0 : 1 that were compared with the experimental values deduced from the fluorescence measurements. The corresponding spots were used to check the consistency of the results. Only experiments showing consistent red, yellow and green spots for ratios of 1 : 0,

Copyright © 2003 John Wiley & Sons, Ltd. 

1 : 1 and 0 : 1, respectively, were kept for subsequent analysis. The final results were expressed as the averages of the ratios between the intensities of 'wounded cells' over the intensities of 'non-wounded' cells.

### Results and discussion

*In vitro* wound healing has been extensively studied after a mechanical injury using a scalpel on confluent monolayers of cultured cells (scratch technique). Until now, the pitfalls of this approach have been a percentage of injured cells too low for quantitative measurements, and the difficulty of obtaining a perfectly reproducible wound. We used a spiral scarificator (Turchi *et al.*, 2002), which allows the wounding of a larger number of cells (30–40%) compared to the classical scratching methods (5%). Moreover, the wounding was perfectly automated and reproducible. After wounding, total RNAs were extracted at different times, and analysed on a DNA microarray. We used for our study 'laboratory-made' arrays containing 1000 genes corresponding to confirmed human transcripts. In order to distinguish truly expressed genes from stochastic fluctuations, the averages of three independent assays were combined. The relative expression of each gene was expressed as the average of the ratios of the normalized measured intensity for wounded cells vs. the intensity measured for control 'non-wounded' cells. For closely related samples, such as in our wounded vs. non-wounded experiments, gene expression should remain globally unaffected between the two conditions (Whiteley *et al.*, 2001). Accordingly, the different distributions of the ratios were centred around 1, over the entire time-course of the experiment (Figure 1). In an initial attempt to check the prediction power of the microarray technique in this model, we focused our attention on the genes displaying the largest variations. Depending on the slides, genes with ratios above 1.7–2.5 or below 0.4–0.6 were subsequently analysed.

Table 1 shows the list of genes that were modulated in keratinocytes after injury. Interestingly, the chromosomal localization of EGR1 on chromosome 5q, of CTGF on chromosome 6q and of DMTF1 on chromosome 7q were close to the chromosomal localizations of TCF7, TNFAIP3 and SERPINE1, respectively, and the pairs of transcripts exhibited parallel changes in expression. These results could be explained by the proposal that enhancers, locus control regions and silencers can alter the expression of multiple genes within regions of the genome (Lee and Young, 2000). The MAPK pathways have been established as the major signalling modules through which cells transduce extracellular signals. Immediate early transcription genes, such as *c-Fos*, *c-Jun* and *Egr1*, are targets for induction and/or activation by MAPK and could mediate the nuclear response to the injury stimulus. *Fos* induction in response to injury has been described in a broad range of tissues and may be an indicator of early MAPK pathway activation. Indeed, Figure 2A shows a transient 14-fold induction of *c-Fos* mRNA 30 min after wounding, that decreases rapidly by more than 90% after 1 h. The quantity of *Egr1* mRNA also increased more than a factor of four, with a similar time-course (Figure 2A). In contrast, the expression *of c-Jun* mRNA was stimulated rapidly after injury (Figure 2A) but its stimulation was

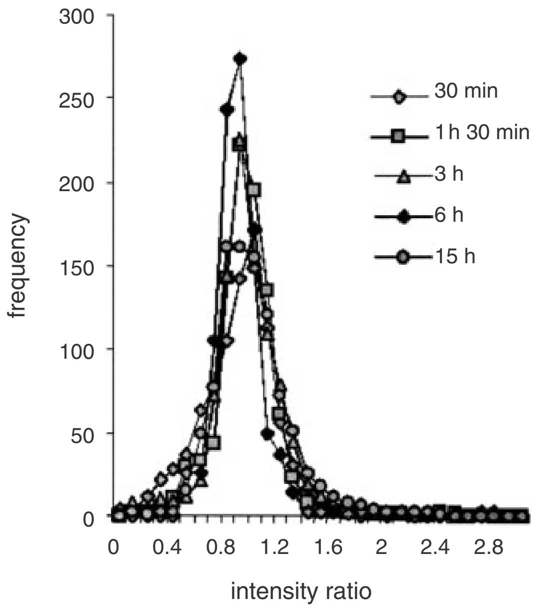

**Figure 1.** Distribution of the intensity ratios for each time experiment. The frequency of each intensity ratio was expressed for each time experiment (30 min, 1 h 30 s, 3 h, 6 h and 15 h). Each time corresponds to an array. Intensity ratios represent the ratio between the intensities measured for wounded cells vs. the intensities measured for non-wounded cells. For each array, the observed ratios are distributed around one

**Table 1.** Classification of genes involved in the response to wounding

| Chromosomal position | Symbol | Locus link | Wounded/control [min–max]$_0$ | Wounded/control [min–max]$_{SB}$ | Class |
|---|---|---|---|---|---|
| 1p32-p31 | JUN | 3725 | 0.98–1.84 | 0.72–1.84 | A |
| 1p34.3 | MACMARCKS | 65108 | 0.51–1.1 | 0.69–1.76 | B |
| 1p36 | EPHA2 | 1969 | 0.71–1.75 | 0.68–1.92 | C |
| 1p36.2 | SLC2A5 | 6518 | 0.34–1.63 | 0.62–1.76 | B |
| 1q21 | MCL1 | 4170 | 0.83–2.10 | 0.75–2.43 | D |
| 1q22-q23 | CD1D | 912 | 0.77–1.68 | 0.69–3.14 | C |
| 1q32.2 | ELF3 | 1999 | 0.57–1.46 | 0.58–1.81 | A |
| 1q42-q43 | KCNK1 | 3775 | 0.53–1.70 | 0.72–1.67 | B |
| 2p22-p21 | LTBP1 | 4052 | 0.55–1.26 | 0.59–1.65 | E |
| 2q11-q14 | SLC20A1 | 6574 | 0.72–1.84 | 0.67–1.58 | B |
| 2q14 | IL1A | 3552 | 0.72–1.60 | 0.41–1.70 | E |
| 2q33-q34 | CASP8 | 841 | 0.87–1.78 | 0.65–2.00 | D |
| 2q37 | ATSV | 547 | 0.91–2.95 | 1.14–4.00 | F |
| 3q28-q29 | CLDN1 | 9076 | 0.78–1.59 | 1.40–6.16 | C |
| 4q13-q21 | IL8 | 3576 | 0.83–1.77 | 0.71–3.58 | E |
| 5q13.1 | OCLN | 4950 | 0.57–1.39 | 0.75–2.17 | C |
| 5q31.1 | EGR1 | 1958 | 0.58–4.47 | 0.25–1.67 | A |
| 5q31.1 | TCF7 | 6932 | 0.42–1.23 | 0.68–2.04 | A |
| 6p24.1 | EDN1 | 1906 | 0.83–1.32 | 0.69–2.33 | E |
| 6q23.1 | CTGF | 1490 | 0.80–1.64 | 0.65–1.82 | E |
| 6q23.1-q25.3 | TNFAIP3 | 7128 | 0.67–4.63 | 0.74–2.46 | A |
| 7q21 | DMTF1 | 9988 | 0.88–2.17 | 0.66–3.60 | A |
| 7q21.3-q22 | SERPINE1 | 5054 | 0.79–1.51 | 0.47–3.26 | G |
| 8q21.2 | E2F5 | 1875 | 0.36–1.20 | 0.52–1.83 | A |
| 8q22.2 | TIEG | 7071 | 0.34–1.13 | 0.88–2.80 | A |
| 10q24 | PLAU | 5328 | 0.82–2.36 | 1.00–3.17 | G |
| 11q23 | CD3D | 915 | 0.53–1.19 | 0.57–2.02 | C |
| 14q24.3 | FOS | 2353 | 0.72–13.56 | 0.56–1.40 | A |
| 17q11.2-q12 | SCYA5 | 6352 | 0.63–2.14 | 0.73–2.19 | E |
| 17q23.2 | ZNF161 | 7716 | 0.64–2.83 | 0.52–1.89 | A |
| 18q21.3 | SERPINB2 | 5055 | 1.23–3.70 | 1.28–13.76 | G |
| 19q13.1 | ZFP36 | 7538 | 0.71–3.31 | 0.69–2.67 | A |
| 20 | TCEA2 | 6919 | 0.54–2.07 | 0.58–2.01 | H |
| 20p12-p11.2 | SNAP25 | 6616 | 0.61–1.17 | 0.68–2.12 | B |
| 22q13.1 | HMOX1 | 3162 | 0.65–2.06 | 0.78–1.95 | B |
| Xp22.3 | IL3RA | 3563 | 0.32–1.80 | 0.50–1.83 | C |
| Unknown | FGF16 | 8823 | 0.55–1.09 | 0.55–3.00 | E |

**Symbols: JUN**, v-jun oncogene; **MACMARCKS**, macrophage myristoylated alanine –rich C kinase substrate; **SLC2A5**, solute carrier family 2, member 5; **MCL1**, myeloid cell leukemia sequence 1; **CDID**, CDID antigen; **ELF3**, E74-like factor 3 (ets-domain transcription factor, epithelial-specific); **KCNK1**, potassium channel, subfamily **K**, member 1; **LTBP1**, latent transforming growth factor $\beta$ binding protein 1; **SLC20A1**, solute carrier family 20, member 1; **IL1A**, interleukin 1 $\alpha$; **CASP8**, caspase 8, apoptosis-related cysteine protease; **ATSV**, axonal transport of synaptic vesicles; **CLDN1**, claudin 1; **IL8**, interleukin 8; **OCLN** occludin; **EGR1**, early growth response 1; **TCF7**, transcription factor 7; **EDN1**, endothelin 1; **CTGF** connective tissue growth factor; **TNFAIL3**, tumour necrosis factor, $\alpha$-induced protein 3; **DMTF1**, cyclin D binding myb-like transcription factor 1; **SERPINE1**, serine (or cysteine) proteinase inhibitor, clade E; **E2F5**, E2F transcription factor 5; **TIEG**, TGF$\beta$-inducible early growth response; **PLAU**, plasminogen activator, urokinase; **CD3D**, CD3D antigen; **FOS**, v-fos oncogene; **SCYA5**, small inducible cytokine A5 (RANTES); **ZNF161**, zinc finger protein 161; **SERPINB2**, serine (or cysteine) proteinase inhibitor, clade B; **ZFP36**, zinc finger protein 36; **TCEA2**, transcription elongation factor A, 2; **SNAP25**, stnaptosomal-associated protein; **HMOX1**, heme oxygenase (decycling) 1; **IL3RA**, interleukin 3 receptor, $\alpha$; **FGF16:** fibroblast growth factor 16.

[min–max]$_0$, minimum and maximum average ratios obtained for wounded keratinocytes in the absence of SB203580;[min-max]$_{SB}$, minimum and maximum average ratios obtained for wounded keratinocytes in the presence of SB203580. Ratios correspond to the normalized intensities of wounded cells vs. control non-wounded cells. For each time point, the value is an average of three independent measurements. The minimum (respectively the maximum) represents the minimal (respectively the maximum) average ratio observed over time (the different time points being 0.5H, 1.5H, 3H, 6H and 15H).

**Class:A**, transcription factor; **B**, intracellular modulators/ion channels/signal transducers; **C**, receptors/cell surface proteins; **D**, cell cycle regulators; **E**, growth factors and cytokines; **F**, cytoskeletal components; **G**, proteases/protease inhibitors; **H**, DNA Synthesis/modification/transcription and nucleotide metabolism. The entire data set is available at: **http://medlab.ipmc.cnrs.fr/publications/Dayem2002_Comparative&Functional_Genomics**

Copyright © 2003 John Wiley & Sons, Ltd. 

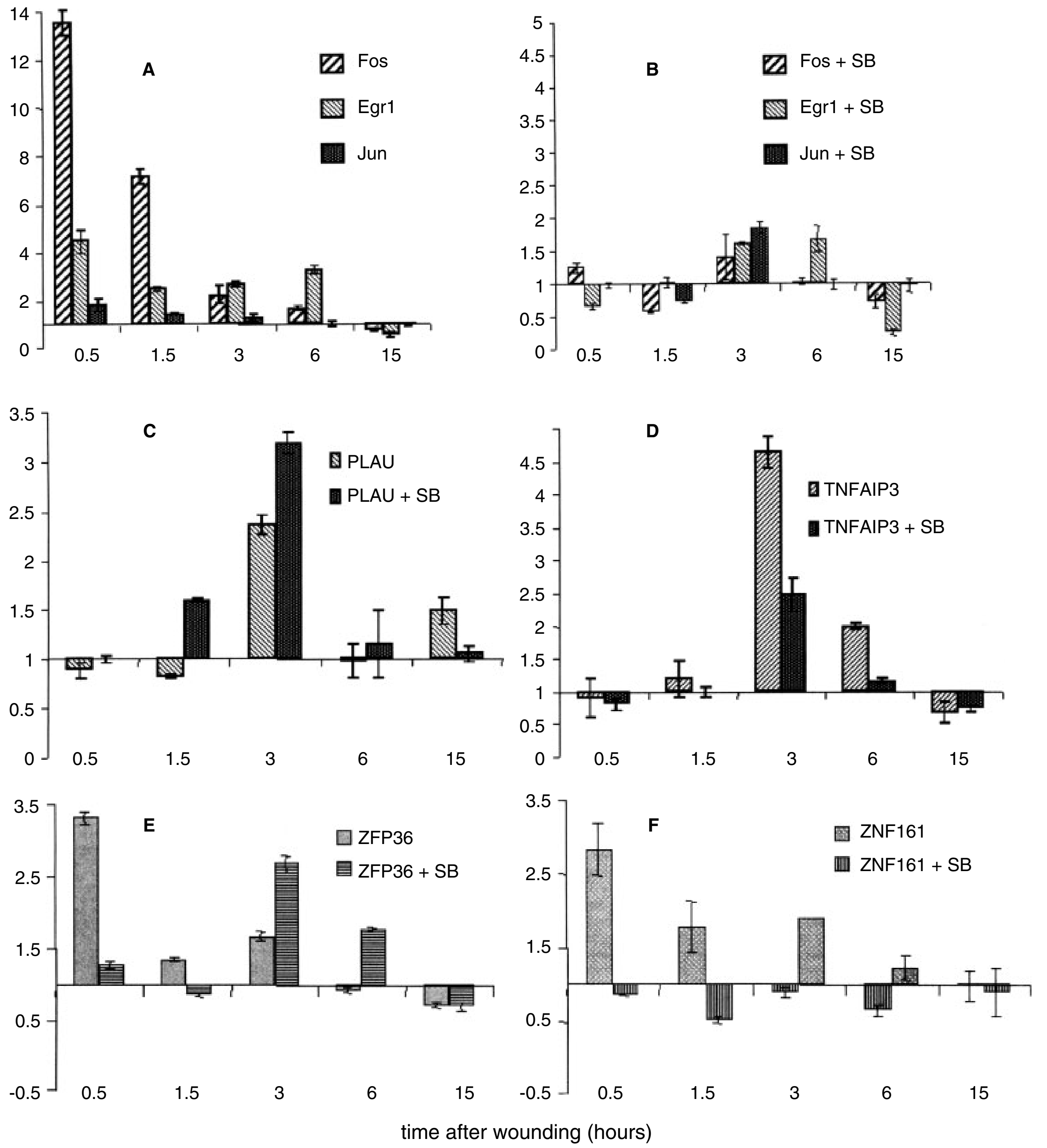

**Figure 2.** Time series of the transcriptional expression of *c-fos, c-jun, Egr1, PLAU, TNFAIP3, Zfp36 and ZNF161* in wounded human keratinocytes, in the presence or absence of SB203580. Human keratinocytes grown as confluent monolayers were wounded using a spiral scarificator. At different times (30 min, 1 h 30 s, 3 h, 6 h, 15 h) after wounding, total RNA was extracted, reverse transcribed in the presence of a fluorophore, and the labelled cDNAs were hybridized on a DNA microarray. Intensity ratios correspond to the normalized fluorescence intensities measured in wounded (W) vs. control non-wounded (NW) keratinocytes. For the experiments performed in the presence of the p38 inhibitor SB203580, cells were preincubated in 30 μM SB203580 2 h before wounding

Copyright © 2003 John Wiley & Sons, Ltd. 

not transient and lasted for at least an additional 4 h, in total accordance with results published by Turchi *et al.*

Mechanical damage created by injury leads to the activation of stress kinases such as p38 and JNK. To examine the role of p38MAPK on gene expression after injury, cells were wounded in the presence of a 30 μM concentration of a p38-specific, cell-permeable inhibitor SB203580 (Lee *et al.*, 1994). The $IC_{50}$ of p38 MAPK inhibition by SB203580 *in vitro* is approximately 0.6 μM (Lee *et al.*, 1994): a 30 μM concentration of SB203580 therefore totally inhibits the enzyme. In the presence of the inhibitor, the wound-healing process was delayed (data not shown) and several genes were inhibited, including *c-Fos, Egr1* and *c-Jun*, known to be controlled by the p38 pathway (Figure 2B). This selective inhibition proves the validity of our microarray data analysis.

Because these initial results were consistent with previous observations by Turchi *et al.*, we then analysed the effect of SB203580 on the expression pattern of other altered genes, which might also be involved into the wound-healing process. Among the other altered genes, we identified many genes either directly controlled by Egr1, or displaying zinc-finger domains like Egr1. Many genes suspected to be relevant for tissue repair contain nucleotide recognition elements for Egr1, such as the transforming growth factor-$\beta$1 (TGF$\beta$1; Kim *et al.*, 1994; Dey *et al.*, 1994), the urokinase-type plasminogen activator PLAU (uPA; Verde *et al.*, 1988), the intercellular adhesion molecule-1 (ICAM-1; Maltzman *et al.*, 1996) and the platelet-derived growth factor (PDGF; Khachigian *et al.*, 1996; Silverman *et al.*, 1997). PLAU possibly participates in wound repair, cell adhesion and cell growth. Gene inactivation experiments demonstrated that the wound-healing process was markedly inhibited in PLAU-/- mice (Romer *et al.*, 1996). Our results show that PLAU expression was increased 2.5-fold 3 h after the wound and was further stimulated by SB203580 (Figure 2C), indicating that p38 is not directly involved in the control of PLAU expression. Interestingly, other experiments showed that the inhibition of p38 by SB203580 strongly potentiated the activity of ERK1/2 stimulated by injury (data not shown). We conclude from these experiments that, in response to injury, the p38 cascade antagonizes the ERK pathways. The higher level of expression of PLAU in SB203580-treated cells (3.5-fold vs. 2.5-fold at 3 h) is therefore consistent with the potentiation of ERK activity.

Like Egr1, TNFAIP3 (also known as ZFP-A20), the tumour necrosis factor alpha-induced protein 3, has zinc finger domains. TNFAIP3 was upregulated approximately five-fold 3 h after injury. More than 60% of the effect was damped in the presence of SB203580 (Figure 2D), indicating that TNFAIP3 can be directly activated by p38MAPK. However, a two-fold stimulation was still observed 3 h after inhibition of p38, suggesting that other pathway(s) was(were) involved in the control of TNFAIP3 expression in healing cells.

Two zinc finger proteins, ZFP36 and ZNF161, were also rapidly induced by the wound (Figure 2E, F). ZNF161 has been suspected to participate in the cellular defence response, but not ZFP36. No direct evidence has yet been provided for the role of these two proteins in response to wounding. When the p38 inhibitor was present, the early induction was totally inhibited, and induction of these two genes took place later (3 h after wounding). These results indicate the role of the p38 cascade for the early induction, but also suggest the existence of a surrogate activating pathway.

The STAT factors (STAT1-6) are a family of signal transducers and activators of transcription (Takeda and Akira, 2000). We also showed that, in wounded keratinocytes, only STAT3 was induced two-fold 90 min after wounding (Figure 3). This information was in total agreement with data indicating that STAT3 inactivation altered skin remodelling (Sano *et al.*, 1999).

When cells were treated with SB203580, STAT3 was totally repressed, indicating that p38 is involved in its induction. In contrast, STAT1, 4 and 6 were not stimulated by injury, but were induced 3 h after wounding only in the presence of SB203580 (Figure 3). p38 therefore exerts a negative effect on the pathway controlling the expression of STAT 1, 4 and 6 in wounded keratinocytes.

The data obtained in the present study validate the complex experimental line that we had to develop in order to analyse DNA microarrays. The good correlation existing between microarray results and reference transcripts (c-fos, c-jun, PLAU, STAT3, EGR1) is a good indication that our procedures for experimental design of the probes, production of the PCR fragments, coating, printing and post-processing of the microarrays, labelling,

Copyright © 2003 John Wiley & Sons, Ltd. 

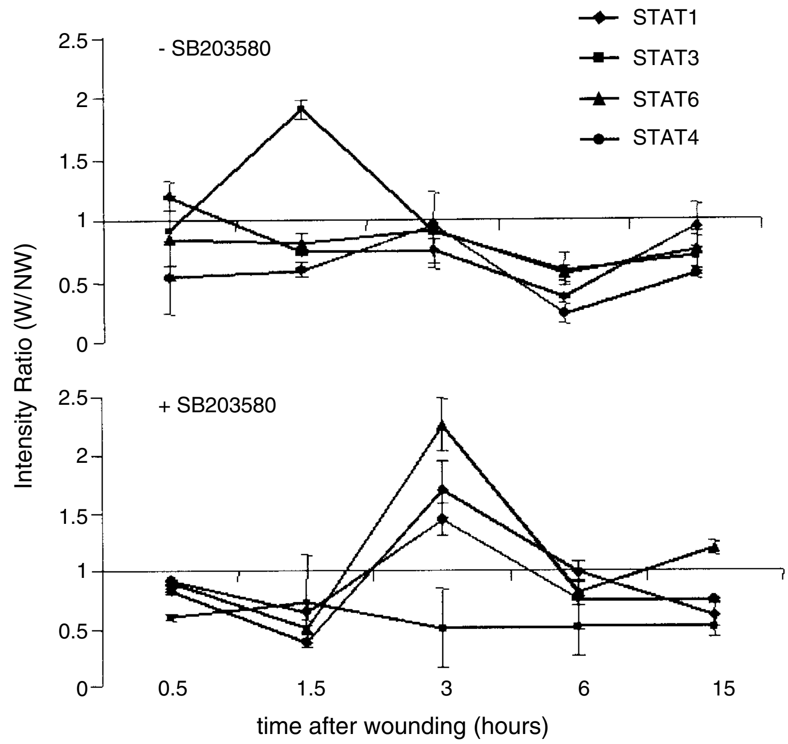

**Figure 3.** Involvement of the STAT factors in the transcriptional response to wounding, in the presence or absence of SB203580. Confluent human keratinocytes were wounded using a spiral scarificator. In experiments performed in the presence of the inhibitor of p38 MAPK, the cells were preincubated with 30 μM SB203580 2 h before wounding. At different times (30 min, 1 h, 3 h, 6 h, 15 h) after wounding, total RNA was extracted, and hybridized on a DNA microarray. STAT1, STAT3, STAT4 and STAT6 expression is represented as the ratios of the normalized fluorescence intensities measured in wounded (W) vs. control non-wounded (NW) keratinocytes hybridizations and quantification, are valid. From a biological point of view, the experiments described herein confirm known patterns of gene expression during the early steps of wound healing, but they also provide original information concerning genes that had never been related to this process, such as TNFAIP3, ZFP36 or ZNF161 (see also Table 1). From similar time courses of expression in the presence or absence of SB203580, close and complementary roles during the early steps of wound healing, or a close genomic association, can probably be inferred for previously unrelated genes.

### Acknowledgements

This work was supported by grants from the CNRS, the GIP Aventis (FRHMR2/9936), the Association pour la Recherche sur le Cancer (No. 9081), the Association Vaincre la Mucoviscidose, the French Ministry for Research, and the Fondation pour la Recherche Médicale (FRM20000374/02). The authors would like to thank Nathalie Scrima and Luc Sofer for excellent technical assistance.

### References

Aronson D, Wojtaszewski JF, Thorell A, *et al.* 1998. Extracellular-regulated protein kinase cascades are activated in response to injury in human skeletal muscle. *Am J Physiol* **275**: C555–C561.

Dey BR, Sukhatme VP, Roberts AB, *et al.* 1994. Repression of the transforming growth factor b1 gene by the Wilms' tumor suppressor WT1 gene product. *Mol Endocrinol* **8**: 595–602.

Dieckgraefe BK, Weems DM, Santoro SA, *et al.* 1997. ERK and p38 MAP kinase pathways are mediators of intestinal epithelial wound-induced signal transduction. *Biochem Biophys Res Commun* **233**: 389–394.

Goke M, Kanai M, Lynch-Devaney K, *et al.* 1998. Rapid mitogen-activated protein kinase activation by transforming growth factor alpha in wounded rat intestinal epithelial cells. *Gastroenterology* **114**: 697–705.

Grembowicz KP, Sprague D, McNeil PL. 1999. Temporary disruption of the plasma membrane is required for c-fos expression in response to mechanical stress. *Mol Biol Cell* **10**: 1247–1257.

Grose R, Harris BS, Cooper L, *et al.* 2002. Immediate early genes *krox-24* and *krox-20* are rapidly upregulated after wounding in the embryonic and adult mouse. *Dev Dyn* **233**: 371–378.

Jaakkola P, Kontusaari S, Kauppi T, *et al.* 1998. Wound re-epithelialization activates a growth factor-responsive enhancer in migrating keratinocytes. *FASEB J* **12**: 959–969.

Kerr MK, Churchill GA. 2001. Experimental design for gene expression microarrays. *Biostatistics* **2**: 183–201.

Khachigian LM, Lindner V, Williams AJ, *et al.* 1996. Egr1-induced endothelial gene expression: a common theme in vascular injury. *Science* **271**: 1427–1431.

Kim SJ, Park K, Rudkin BB, *et al.* 1994. Nerve growth factor induces transcription of transforming growth factor $\beta$1 through a specific promoter element in PC12 cells. *J Biol Chem* **269**: 3739–3744.

Lee JC, Laydon JT, McDonnell PC, *et al.* 1994. A protein kinase involved in the regulation of inflammatory cytokine biosynthesis. *Nature* **372**: 739–746.

Lee DJ, Rosenfeldt H, Grinnell F. 2000. Activation of ERK and p38 MAP kinases in human fibroblasts during collagen matrix contraction. *Exp Cell Res* **257**: 190–197.

Lee TI, Young RA. 2000. Transcription of eukaryotic protein-coding genes. *Ann Rev Genet* **34**: 77–137.

Maltzman JS, Carmen JA, Monroe JG. 1996. Transcriptional regulation of the Icam-1 gene in antigen receptor- and phorbol ester-stimulated B lymphocytes: role for the transcription factor Egr1. *J Exp Med* **183**: 1747–1759.

Martin P, Nobes CD. 1992. An early molecular component of the wound healing response in rat embryos — induction of the c-fos protein in cells at the epidermal wound margin. *Mech Dev* **38**: 209–215.

Meloche S, Seuwen K, Pages G, *et al.* 1992. Biphasic and synergistic activation of p44mapk (ERK1) by growth factors: correlation between late phase activation and mitogenicity. *Mol Endocrinol* **6**: 845–854.

Rheinwald JG, Green H. 1975. Formation of a keratinizing epithelium in culture by a cloned cell line derived from a teratoma. *Cell* **6**: 331–343.

Romer J, Bugge TH, Pyke C, *et al.* 1996. Impaired wound healing in mice with a disrupted plasminogen gene. *Nature Med* **2**: 287–292.

Sano S, Itami S, Takeda K, *et al.* 1999. Keratinocyte-specific ablation of Stat3 exhibits impaired skin remodeling, but does not affect skin morphogenesis. *EMBO J* **18**: 4657–4668.

Seo S, Okamoto M, Seto H, *et al.* 1995. Tobacco MAP kinase: a possible mediator in wound signal transduction pathways. *Science* **270**: 1988–1992.

Silverman ES, Khachigian LM, Lindner V, *et al.* 1997. Inducible PDGF A-chain transcription in smooth muscle cells is mediated by Egr-1 displacement of Sp1 and Sp3. *Am J Physiol* **273**: H1415–H1426.

Takeda K, Akira S. 2000. STAT family of transcription factors in cytokine-mediated biological responses. *Cytokine Growth Factor Rev* **11**: 199–207.

Turchi L, Chassot A, Rezzonico R, *et al.* 2002. Dynamic characterization of the molecular events during *in vitro* epidermal wound healing. *J Invest Dermatol* **179**: 56–63.

Verde P, Boast S, Franze A, *et al.* 1988. An upstream enhancer and a negative element in the 5′ flanking region of the human urokinase plasminogen activator gene. *Nucleic Acids Res* **16**: 10 699–10 716.

Verrier B, Muller D, Bravo R, *et al.* 1986. Wounding a fibroblast monolayer results in the rapid induction of the *c-fos* proto-oncogene. *EMBO J* **5**: 913–917.

Whiteley M, Bangera MG, Bumgarner RE, *et al.* 2001. Gene expression in *Pseudomonas aeruginosa* biofilms. *Nature* **413**: 860–864.

Copyright © 2003 John Wiley & Sons, Ltd. 

## References

[PDF_00650] Aronson D., Wojtaszewski J. F., Thorell A., Nygren J., Zangen D., Richter E. A., Ljungqvist O., Fielding R. A., Goodyear L. J. (1998). Extracellular-regulated protein kinase cascades are activated in response to injury in human skeletal muscle.. Am J Physiol.

[PDF_00653] Dey B. R., Sukhatme V. P., Roberts A. B., Sporn M. B., Rauscher F. J., Kim S. J. (1994). Repression of the transforming growth factor-beta 1 gene by the Wilms' tumor suppressor WT1 gene product.. Mol Endocrinol.

[PDF_00656] Dieckgraefe B. K., Weems D. M., Santoro S. A., Alpers D. H. (1997). ERK and p38 MAP kinase pathways are mediators of intestinal epithelial wound-induced signal transduction.. Biochem Biophys Res Commun.

[PDF_00670] Grose Richard, Harris Brett S., Cooper Lisa, Topilko Piotr, Martin Paul (2002). Immediate early genes krox-24 and krox-20 are rapidly up-regulated after wounding in the embryonic and adult mouse.. Dev Dyn.

[PDF_00660] Göke M., Kanai M., Lynch-Devaney K., Podolsky D. K. (1998). Rapid mitogen-activated protein kinase activation by transforming growth factor alpha in wounded rat intestinal epithelial cells.. Gastroenterology.

[PDF_00673] Jaakkola P., Kontusaari S., Kauppi T., Mätä A., Jalkanen M. (1998). Wound reepithelialization activates a growth factor-responsive enhancer in migrating keratinocytes.. FASEB J.

[PDF_00676] Kerr M. K., Churchill G. A. (2001). Experimental design for gene expression microarrays.. Biostatistics.

[PDF_00678] Khachigian L. M., Lindner V., Williams A. J., Collins T. (1996). Egr-1-induced endothelial gene expression: a common theme in vascular injury.. Science.

[PDF_00681] Kim S. J., Park K., Rudkin B. B., Dey B. R., Sporn M. B., Roberts A. B. (1994). Nerve growth factor induces transcription of transforming growth factor-beta 1 through a specific promoter element in PC12 cells.. J Biol Chem.

[PDF_00688] Lee D. J., Rosenfeldt H., Grinnell F. (2000). Activation of ERK and p38 MAP kinases in human fibroblasts during collagen matrix contraction.. Exp Cell Res.

[PDF_00685] Lee J. C., Laydon J. T., McDonnell P. C., Gallagher T. F., Kumar S., Green D., McNulty D., Blumenthal M. J., Heys J. R., Landvatter S. W. (1994). A protein kinase involved in the regulation of inflammatory cytokine biosynthesis.. Nature.

[PDF_00691] Lee T. I., Young R. A. (2000). Transcription of eukaryotic protein-coding genes.. Annu Rev Genet.

[PDF_00693] Maltzman J. S., Carmen J. A., Monroe J. G. (1996). Transcriptional regulation of the Icam-1 gene in antigen receptor- and phorbol ester-stimulated B lymphocytes: role for transcription factor EGR1.. J Exp Med.

[PDF_00697] Martin P., Nobes C. D. (1992). An early molecular component of the wound healing response in rat embryos--induction of c-fos protein in cells at the epidermal wound margin.. Mech Dev.

[PDF_00701] Meloche S., Seuwen K., Pagès G., Pouysségur J. (1992). Biphasic and synergistic activation of p44mapk (ERK1) by growth factors: correlation between late phase activation and mitogenicity.. Mol Endocrinol.

[PDF_00705] Rheinwald J. G., Green H. (1975). Serial cultivation of strains of human epidermal keratinocytes: the formation of keratinizing colonies from single cells.. Cell.

[PDF_00708] Romer J., Bugge T. H., Pyke C., Lund L. R., Flick M. J., Degen J. L., Dano K. (1996). Impaired wound healing in mice with a disrupted plasminogen gene.. Nat Med.

[PDF_00711] Sano S., Itami S., Takeda K., Tarutani M., Yamaguchi Y., Miura H., Yoshikawa K., Akira S., Takeda J. (1999). Keratinocyte-specific ablation of Stat3 exhibits impaired skin remodeling, but does not affect skin morphogenesis.. EMBO J.

[PDF_00714] Seo S., Okamoto M., Seto H., Ishizuka K., Sano H., Ohashi Y. (1995). Tobacco MAP kinase: a possible mediator in wound signal transduction pathways.. Science.

[PDF_00717] Silverman E. S., Khachigian L. M., Lindner V., Williams A. J., Collins T. (1997). Inducible PDGF A-chain transcription in smooth muscle cells is mediated by Egr-1 displacement of Sp1 and Sp3.. Am J Physiol.

[PDF_00721] Takeda K., Akira S. (2000). STAT family of transcription factors in cytokine-mediated biological responses.. Cytokine Growth Factor Rev.

[PDF_00724] Turchi Laurent, Chassot Anne Amandine, Rezzonico Roger, Yeow Karen, Loubat Agnès, Ferrua Bernard, Lenegrate Gaëlle, Ortonne Jean Paul, Ponzio Gilles (2002). Dynamic characterization of the molecular events during in vitro epidermal wound healing.. J Invest Dermatol.

[PDF_00731] Verrier B., Müller D., Bravo R., Müller R. (1986). Wounding a fibroblast monolayer results in the rapid induction of the c-fos proto-oncogene.. EMBO J.

[PDF_00734] Whiteley M., Bangera M. G., Bumgarner R. E., Parsek M. R., Teitzel G. M., Lory S., Greenberg E. P. (2001). Gene expression in Pseudomonas aeruginosa biofilms.. Nature.

